# Supplementary material for: DLMM as a lossless one-shot algorithm for collaborative multi-site distributed linear mixed models
Source: Nat Commun. 2022 Mar 30;13:1678. doi: 10.1038/s41467-022-29160-4 (PMC8967932; doi:10.1038/s41467-022-29160-4)
Supplement: Supplementary file 1 — Supplementary Information [file 41467_2022_29160_MOESM1_ESM.pdf]

Supplementary Information for "DLMM -- A Lossless One-shot Algorithm of Distributed Linear Mixed Models for Collaborative Multi-site Modeling"

Supplementary Notes

COVID-19 hospitalization length of stay study using UHG data

The detailed cohort inclusion criteria is shown in a flowchart in Figure S1. We include individuals at least 18 years of age, with at least 6 months of enrollment in Medicare Advantage from January through December 2019 and available claims data. We include COVID-19 patients who did not have a discharge status of "expired" prior to September 30, 2020 and consider sites with at least 30 cases of COVID-19 hospitalization. The COVID-19 inpatient case distribution by state is shown in Figure S2.

We analyze the association between COVID-19 patients' hospitalization LOS and various demographic variables and comorbidities by the linear mixed model. The covariates include demographic variables such as age, gender, and race, and comorbidities such as cancer, chronic obstructive pulmonary disease (COPD), heart disease, hypertension, hyperlipidemia, kidney disease, obesity and Charlson comorbidity index (CCI) score. We provide the details of the ICD-10 codes used to define the comorbidities and CCI score in Table S1. For the sake of interpretability, we categorize age into three categories as 18-65, 65-80, and $\geq$80 and set 18-65 as the reference category. Similarly, CCI scores are classified into three categories as 0-1, 2-4, and $\geq$5 with 0-1 as the reference category. We present the summary of the variables in Table S2.

We assume site-specific random intercepts and test the significance of random effects of each individual covariate univariately. We select the covariates for which the corresponding p-value $\leq$0.05. In particular, we select random slopes for obesity, diabetes, and hyperlipidemia. A LMM with random intercept and random effects for obesity, diabetes, and hyperlipidemia is then fitted

by either pooling the IPD together, or the proposed DLMM algorithm. We also calculate the BLUPs and the prediction intervals of the random effects at each site by (11). The results comparing the two approaches are presented in Figure S3.

International COVID-19 hospital LOS study

Below is a description of the actual workflow of conducting the international COVID-19 length of stay study in the OHDSI network. See also Figure S4 for a visualization.

1. Project Initiation – Protocol Development: the project leaders initiate a project and develop an analysis protocol (attached at the end of this response letter, and also submitted as a research supplementary material in our submission of revision). In addition to the analysis protocol, the project leaders need to prepare and test computing programs (e.g., R programs) that are ready for the participating sites to run at their local site (usually data in OMOP CDM) with results prepared in the right format;
2. Recruitment of participating sites: the project leaders post the research project to the OHDSI forum (currently in Microsoft Team) with a deadline of 2-3 weeks for the participants to comment, ask questions, and join this project;
3. Communication: the project leaders create an email list or SSH File Transfer Protocol (sftp) file sharing platform with the contact persons from all participating sites, distribute the prepared R program, and set a deadline for returning the results (of aggregated data) (usually 4 weeks). During these four weeks, the participating sites can ask questions that they encounter during the application of the algorithms. At the end of four weeks, the participating sites share all the requested results (i.e., aggregated data specified by the algorithm);
4. Aggregation of multi-site results and submitting the final results: the project leaders conduct the final analyses by aggregating the results from all participating sites, and share the results to all participants in a written manuscript for comments before submission.

The 11 collaborative databases are listed as below, also see Supplementary Table 3.

- UnitedHealth Group (**UHG**) Clinical Discovery Portal (also analyzed in Section 2.1): A database of medical claims and hospitalizations from a national claims data warehouse in the United States.
- **OneFlorida** data: A CRN that contains robust and longitudinal patient-level linked EHR and claims data from approximately 15 million Floridians from 12 unique healthcare organizations.
- The Stanford Medicine Research Data Repository (**STARR**) [1]: An EHR database of approximately three million patients from Stanford Hospitals and Clinics and the Lucile Packard Children’s Hospital in the United States.
- Columbia University Irving Medical Center Data Warehouse (**CUIMC**): Columbia University EHR database containing records from hospitals in New York City.
- IBM MarketScan Commercial Database (**CCAE**): A database of health insurance claims from large employers and health plans who provide private healthcare coverage to employees, their spouses, and dependents in the United States. The patients are younger than 65.
- IBM MarketScan Medicare Supplemental Database (**MDCR**): A database of health insurance claims representing retirees (aged 65 or older) in the United States with primary or Medicare supplemental coverage through privately insured fee-for-service, point-of-service, or capitated health plans.
- Optum de-identified Electronic Health Record Dataset (**Optum EHR**): A database of electronic healthcare records for patients in the United States.
- **Optum COVID** data sets [2]: The data are sourced from Optum’s longitudinal EHR repository derived from more than 700 hospitals and 7,000 clinics, including patients who have documented clinical care from January 2007 through to the most current monthly

data release with a documented diagnosis of COVID-19 or acute respiratory illness after February 1, 2020 and/or documented COVID-19 testing.

- Tufts Medical Center Research Data Warehouse (**TRDW**): EHR database containing records from Tufts Medical Center, Tufts Children's Hospital, and associated primary and tertiary care clinics fused with oncology data from the Tufts MC Tumor Registry, and death data from the Massachusetts State Registry of Vital Records and Statistics.
- The Information System for Research in Primary Care (**SIDIAP**): An EHR database containing primary care records partially linked to inpatient data representing 80% of the general population in Spain-Catalonia.
- Health Insurance and Review Assessment COVID database (**HIRA COVID**) [3]: A national health insurance claims database in South Korea including all patients who suspected or confirmed as COVID-19.

The UHG data: Because no identifiable protected health information was extracted or accessed during the course of the study, and all data were accessed in compliance with the Health Insurance Portability and Accountability Act's rules, Institutional Review Board approval or waiver of authorization was not required. The official exemption by the UHG IRB is also available.

The OneFlorida data: At OneFlorida, the access to the HIPAA Limited Data Set was reviewed by the University of Florida Institutional Review Board under IRB202001831. The analysis was run locally at the University of Florida and only summary statistics were shared.

The Optum COVID data (November 2020): Given the urgent need to clinically understand the novel virus of COVID 19, Optum developed a low latency data pipeline that enables minimal data lag, while preserving as much clinical data as possible. The data is sourced from Optum's longitudinal EHR repository, which is derived from dozens of healthcare provider organizations in

the United States, including more than 700 Hospitals and 7000 Clinics. The data is certified as de-identified by an independent statistical expert following HIPAA statistical de-identification rules and managed according to Optum® customer data use agreements[1],[2]. The COVID-19 data asset incorporates a wide swath of raw clinical data, including new, unmapped COVID-specific clinical data points from both Inpatient and Ambulatory electronic medical records (EMRs), practice management systems and numerous other internal systems. Information is processed from across the continuum of care, including acute inpatient stays and outpatient visits. The COVID-19 data captures point of care diagnostics specific to the COVID-19 patient during initial presentation, acute illness and convalescence with over 500 mapped labs and bedside observations, including COVID-19 specific testing.

The Optum COVID-19 data elements include patient level information: demographics, mortality, as well as clinical interventions such as medications prescribed and administered. The Data is comprised of multiple tables that can be linked by a common patient identifier (anonymous, randomized string of characters). The COVID-19 patient base including patients in the Electronic Health Record Database who have documented clinical care from January 2007 through to the most current monthly data release with a documented diagnosis of COVID-19 or acute respiratory illness after 02/01/2020 and/or documented COVID-19 testing (positive or negative result).

The Tufts MC data: The Tufts MC Research Data Warehouse (TRDW) is developed and maintained at the Tufts MC Clinical and Translational Science Institute. It is the merged repository of data from four EHRs used at Tufts Medical Center and Tufts Children's hospital and their

[1] 45 CFR 164.514(b)(1).

[2] Guidance Regarding Methods for De-identification of Protected Health Information in Accordance with the Health Information Insurance Portability and Accountability Act (HIPAA) Privacy Rule (Dated as September 4, 2012, as first released on November 26, 2012).

affiliated specialty and primary care clinics. It contains longitudinal data on approximately one million patients with more complete capture beginning in 2007. It is updated frequently with refresh schedules varying by data source. TRDW ingests data from labs, prescription orders, diagnoses, procedures, a variety of other observations on patients, patient demographics, care providers, care sites, unstructured data sources including notes and notes-derived medical concepts, manually abstracted data from accredited registries such as the Tufts MC Tumor Registry, physiological signal data such as EKG measurements, and recent vital status information from the Massachusetts Registry of Vital Records and Statistics. Addition manual curation of data is done during peak Covid care periods to ensure accurate capture of invasive ventilation and receipt of acute care inside and outside of officially designated intensive care units. Concepts from unstructured data are extracted using either of two robust NLP pipelines (OHNLP and ClarityNLP). All concepts are standardized to the OMOP common data model and its ontologies. The OMOP instance of the TRDW is periodically examined for >3000 data quality checks using the Data Quality Dashboard to facilitate ongoing data quality improvement and transparency with respect to unresolvable issues.

The CUIMC, CCAE, HIRA COVID, MDCR, Optum EHR, SIDIAP, TRDW and STARR data: The Observational Healthcare Data Science & Informatics (OHDSI) collaborative network was used to perform an international study. All data partners within the OHDSI network map their observational data into a Common Data Model (CDM) known as the Observational Medical Outcome Partnership (OMOP) CDM. This enables study analysis scripts to be directly shared. It is not possible to share patient level data across the OHDSI network, but aggregate data results can be readily shared given appropriate IRB approval.

In the OHDSI study patients were included into the cohort if they had an inpatient visit between January 2020 and September 2020 satisfying

- age 18 years or older
- A COVID-19 diagnosis or positive test recorded up to 21 days prior to the visit or during the visit
- Been active in the database for 6 months or more prior to the inpatient visit
- Did not have a discharge status of “expired” prior to September 30, 2020.

A project protocol and an R package were created to implement the study on any suitable database mapped to the OMOP CDM https://github.com/ohdsi-studies/DistributedLMM. Databases in the network that contained COVID-19 hospitalizations ran the study and shared the aggregated data results.

CCAE, MRCR, Optum Claims and Optum EHR we reviewed by the New England Institutional Review Board (IRB) and were determined to be exempt from broad IRB approval, as this research project did not involve human subject research. The use of SIDIAP database was approved by the SIDIAP Scientific Committee and the IDIAPJGol Clinical Research Ethics Committee. CUIMC, TRDW, STARR, and HIRA COVID had institutional review board approval for the analysis, or used deidentified data, and thus the analysis was determined not to be human subjects research and informed consent was not deemed necessary at any site.

The HIRA COVID data: The authors appreciate the health care professionals dedicated to treating patients with COVID-19 in Korea and the Ministry of Health and Welfare and the Health Insurance Review & Assessment Service of Korea for sharing invaluable national health insurance claims data in a prompt manner.

Comparison of the LMM with meta-analysis for the COVID-19 hospitalization LOS study

The linear mixed model (LMM) use in our study is closely connected with the (random-effects) meta-analysis. Debray et al. 2012 [4] used the meta-analysis to synthesize the common effects of predictors and further calibrate local IPD prediction. Specifically, if a (random-effects) meta-analysis approach is used, the BLUP for site-specific random effects could also be estimated for the purpose of site-specific prediction. This is very similar to what LMM aims to do. We thus provide a comparison of LMM with (random-effects) meta-analysis approach for the LOS study, as summarized by Supplementary Figure 5 below. Specifically, we compared three methods:

1) BLUP (meta): best linear unbiased predictor (BLUP) of intercept or a covariate effect using two-stage IPD-meta-analysis;

2) Individual LM est: Estimate based on the data from a given site only;

3) BLUP (LMM): BLUP based on the proposed (one-stage) DLMM algorithm (which is identical to the BLUP based on a LMM).

The results show similar (but not identical) fixed-effects estimates (dotted and dashed horizontal lines), as well as the shrinkage pattern from the individual estimates to the estimated BLUPs based on the BLUP (meta) or BLUP (LMM). We note that overall the estimated BLUPs and the fixed effects are similar, yet such similarity could be dependent on various factors, such as the number of patients per site, the ratio between the within-site heterogeneity and the between-site heterogeneity (which is corresponding to the intra-cluster correlation), and the number of sites.

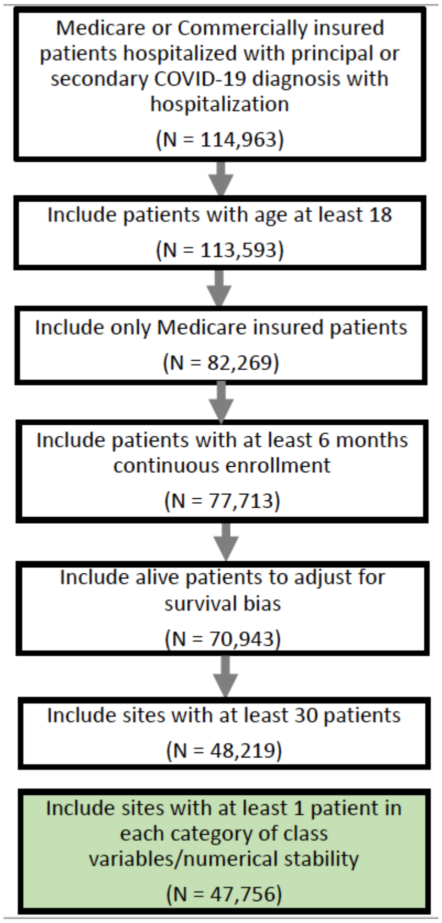

Supplementary Figure 1. Flow chart of cohort definition for the COVID-19 hospitalization length of stay study using UHG claims data.

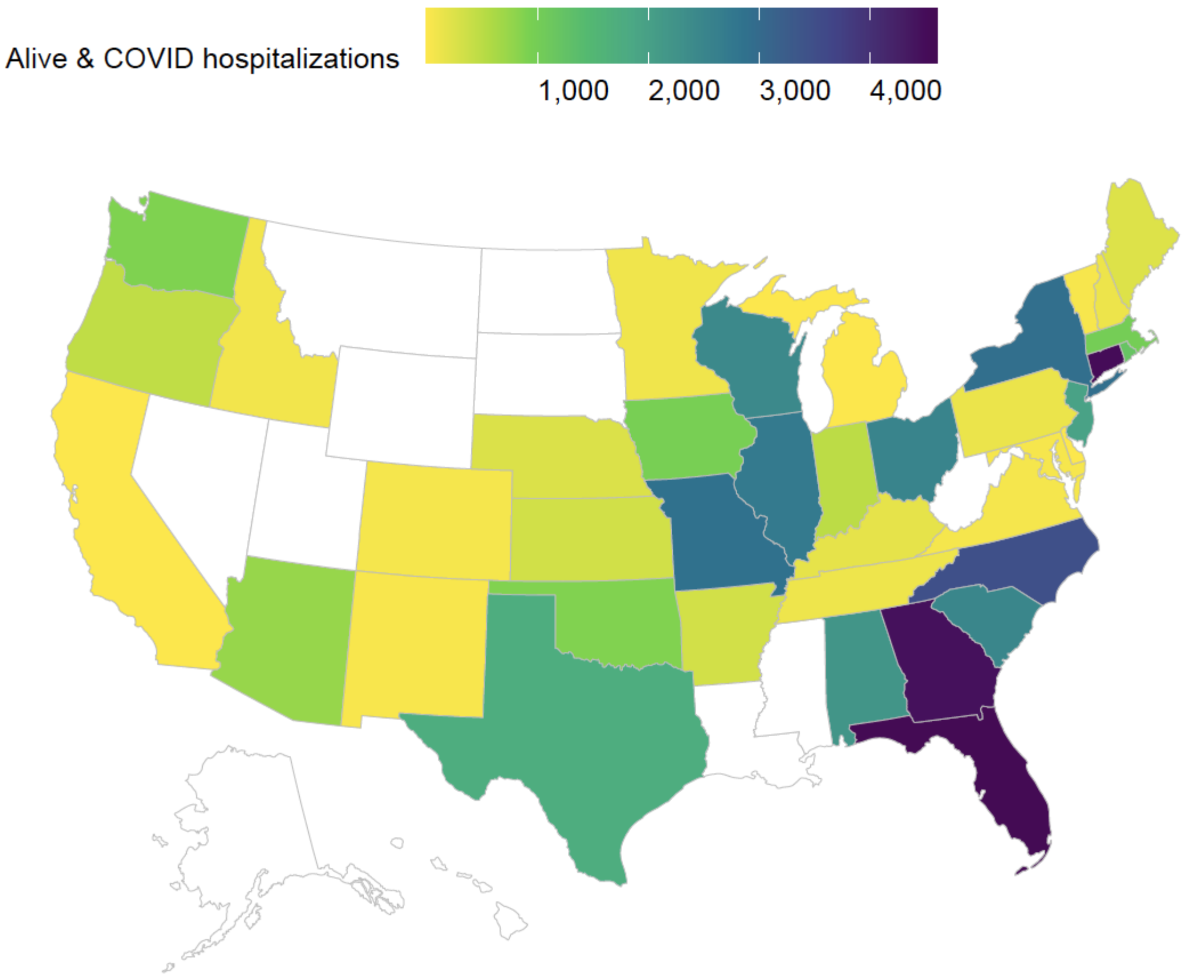

Supplementary Figure 2. COVID-19 inpatient case distribution: number of hospitalizations by state. Data are extracted from UHG medical claims.

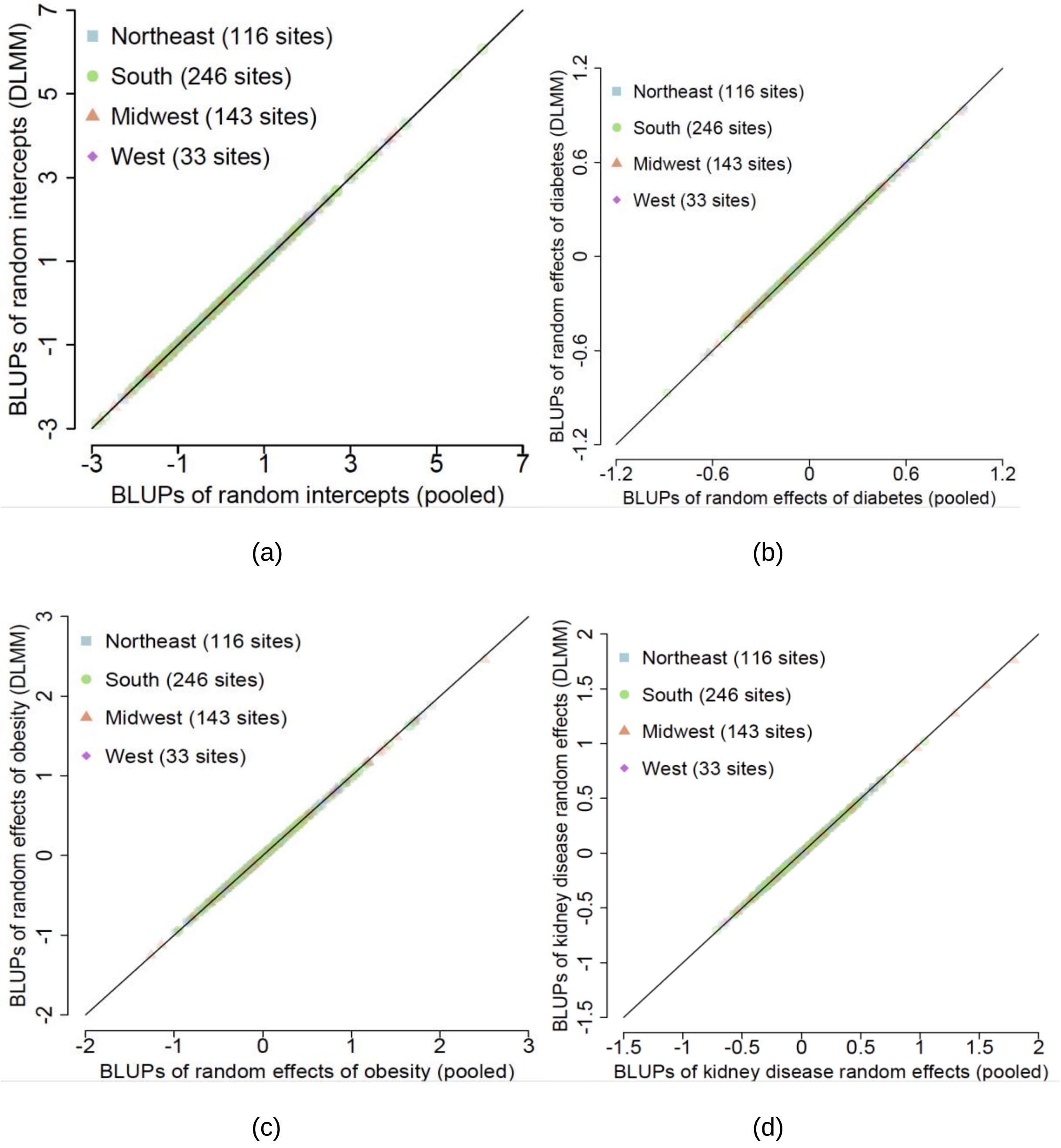

Northeast (116 sites)
South (246 sites)
Midwest (143 sites)
West (33 sites)
BLUPs of random intercepts (DLMM)
BLUPs of random intercepts (pooled)
(a)
BLUPs of random effects of diabetes (DLMM)
BLUPs of random effects of diabetes (pooled)
(b)
BLUPs of random effects of obesity (DLMM)
BLUPs of random effects of obesity (pooled)
(c)
BLUPs of kidney disease random effects (DLMM)
BLUPs of kidney disease random effects (pooled)
(d)

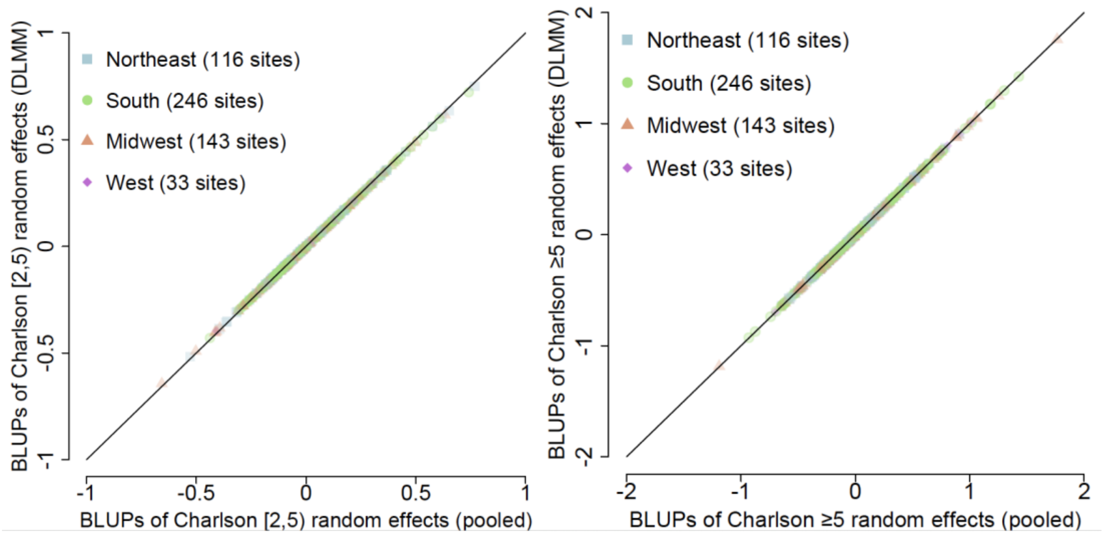

(e) (f)

Supplementary Figure 3. Comparison of the best linear unbiased predictors (BLUPs) of the random effects by pooled and DLMM methods. The BLUPs are obtained from a linear mixed model with COVID-19 hospitalization as the outcome and demographics and comorbidity variables as covariates. Diabetes, obesity, kidney disease and Charlson score are selected as having significant random effects.

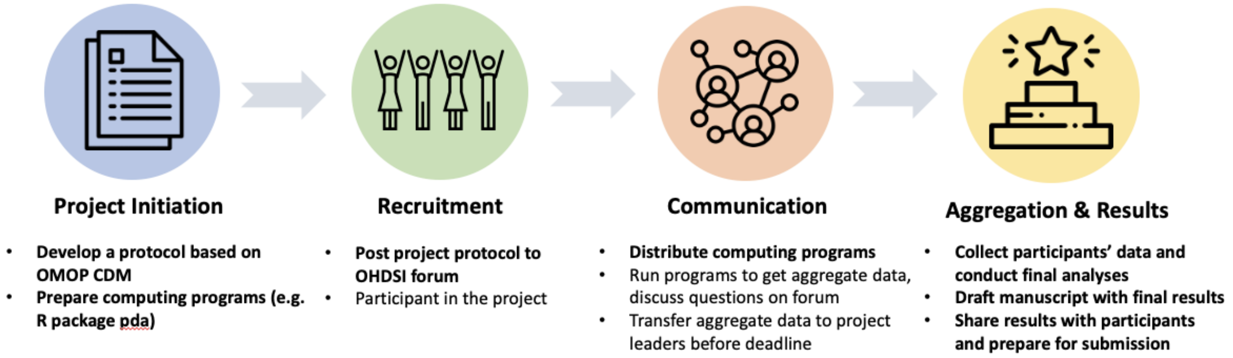

Supplementary Figure 4. Workflow of the collaborative international COVID-19 length of stay study in the OHDSI network. Activities of the project coordinator are shown in bold.

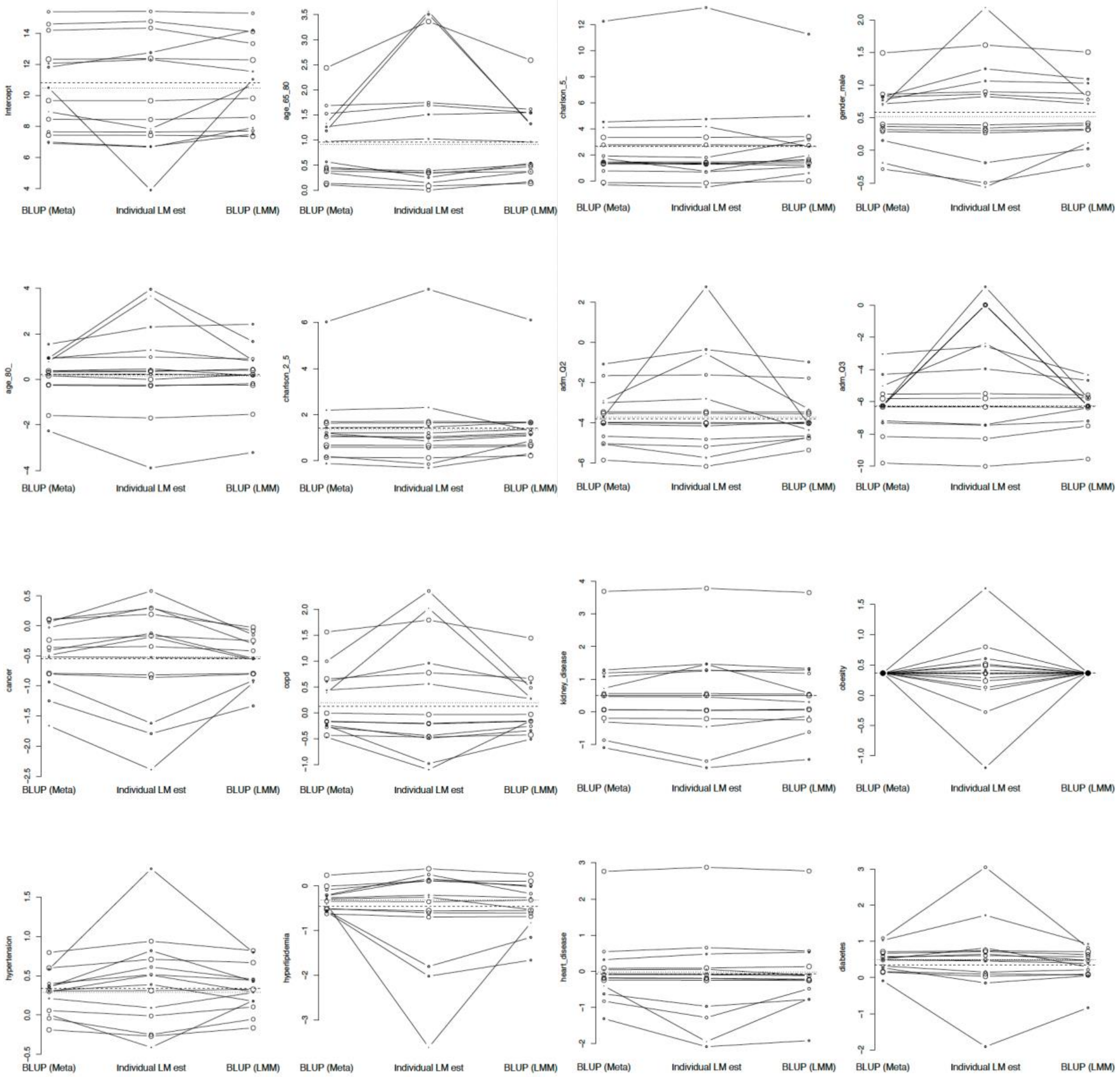

Supplementary Figure 5. The multi-center COVID-19 hospitalization LOS study using three approaches: individual linear model estimates based on the data from a given site only, and BLUP estimates by random-effects meta-analyses or linear mixed model. The dotted and dashed horizontal lines indicate the estimated fixed effects. Both meta-analyses and LMM detect no random effect for obesity on LOS.

Supplementary Table 1. ICD-10-CM codes used to calculate Charlson comorbidity index in the UHG data.

Comorbidity	ICD-10-CM Codes
Acquired immunodeficiency syndrome (AIDS)	B20, B21, B22, B24
Arthritis	M05, M06, M315, M32, M33, M34, M351, M353, M360
Cerebrovascular Disease	G45, G46, H340, I60, I61, I62, I63, I64, I65, I66, I67, I68, I69
Congestive Heart Failure (CHF)	I099, I110, I130, I132, I255, I420, I425, I426, I427, I428, I429, I43, I50, P290
Chronic obstructive pulmonary disease (COPD)	I278, I279, J40, J41, J42, J43, J44, J45, J46, J47, J60, J61, J62, J63, J64, J65, J66, J67, J684, J701, J703
Dementia	F00, F01, F02, F03, F051, G30, G311
Diabetes	E100, E101, E106, E108, E109, E110, E111, E116, E118, E119, E120, E121, E126, E128, E129, E130, E131, E136, E138, E139, E140, E141, E146, E148, E149
Diabetes with complications	E102, E103, E104, E105, E107, E112, E113, E114, E115, E117, E122, E123, E124, E125, E127, E132, E133, E134, E135, E137, E142, E143, E144, E145, E147
Mild Liver Disease	B18, K700, K701, K702, K703, K709, K713, K714, K715, K717, K73, K74, K760, K762, K763, K764, K768, K769, Z944
Moderate/ Severe Liver Disease	I850, I859, I864, I982, K704, K711, K721, K729, K765, K766, K767
Metastatic solid malignancy	C77, C78, C79, C80
Myocardial infarction	I21, I22, I252
Paralysis	G041, G114, G801, G802, G81, G82, G830, G831, G832, G833, G834, G839
Peripheral Vascular Disease	I70, I71, I731, I738, I739, I771, I790, I792, K551, K558, K559, Z958, Z959
Peptic Ulcer Disease	K25, K26, K27, K28
Renal Disease	I120, I131, N032, N033, N034, N035, N036, N037, N052, N053, N054, N055, N056, N057, N18, N19, N250, Z490, Z491, Z492, Z940, Z992
Tumor	C00, C01, C02, C03, C04, C05, C06, C07, C08, C09, C10, C11, C12, C13, C14, C15, C16, C17, C18, C19, C20, C21, C22, C23, C24, C25, C26, C30, C31, C32, C33, C34, C37, C38, C39, C40, C41, C43, C45, C46, C47, C48, C49, C50, C51, C52, C53, C54, C55, C56, C57, C58, C60, C61, C62, C63, C64, C65, C66, C67, C68, C69, C70, C71, C72, C73, C74, C75, C76, C81, C82, C83, C84, C85, C88, C90, C91, C92, C93, C94, C95, C96, C97

Supplementary Table 2. COVID-19 hospitalized patients characteristics in the UHG data.

**Patients Number**		**47756**
**Hospitals by Region, count (%) Total**		538 (100%)
Mid-west		143 (26.6%)
North-east		116 (21.6%)
South		246 (45.7%)
West		33 (6.13%)
**Patient Level Characteristics**		
**Age category, count (%):**	[18, 65)	5638 (11.8%)
	[65, 80)	24571 (51.5%)
	≥ 85	17547 (36.7%)
**Gender, count (%):**	Male (%)	21625 (45.3%)
	Female (%)	26131 (54.7%)
**Race, count (%):**	Non-Hispanic White (%)	35083 (73.5%)
	Other/unknown (%)	12673 (26.5%)
**Charlson Score, count (%):**	[0, 2)	12578 (26.3%)
	[2, 5)	16805 (35.2%)
	≥ 5	18373 (38.5%)
**Comorbidities, count (%):**	Cancer	9612 (20.1%)
	Chronic Obstructive Pulmonary Disease	12035 (25.2%)
	Diabetes	21327 (44.7%)
	Heart disease	28586 (59.9%)
	Hyperlipidemia	28954 (60.6%)
	Hypertension	37986 (79.5%)
	Kidney disease	17664 (36.5%)
	Obesity	5865 (12.3%)
**Patient Outcome: Length of Stay in days, mean (sd)**		(8.6, 11.1)

Supplementary Table 3. Description of the collaborative data sources. The UHG data are divided into four “sites” based on geographical area. The sample sizes are 12,178 for Northeast (UHG.NE), 20,565 for South (UHG.S), 12,717 for Midwest (UHG.MW), and 2,296 for West (UHG.W). Because the UHG dataset encompasses thousands of healthcare provider organizations, there is a possibility of data overlap. We estimate a 14% overlap with other US-based datasets, consistent with UHG’s market share of U.S. health insurance.

	Data set name	Description	Type	Sample size
1	UHG	UnitedHealth Group Clinical Discovery Portal	medical claims	47,756
2	CCAE	IBM MarketScan® Commercial	medical claims	18,185
3	MDCR	IBM MarketScan® Medicare Supplemental Database	medical claims	4,024
4	Optum COVID	Processed by UT Health Center at Houston	medical claims	14,139
5	OneFlorida	OneFlorida system (6 hospitals)	EHR and medical claims	2,626
6	STARR	The Stanford Medicine Research Data Repository	EHR	995
7	CUIMC	Columbia University Irving Medical Center	EHR	3,289
8	Optum EHR	Optum® de-identified Electronic Health Record Dataset COVID	EHR	6,114
9	TRDW	Tufts Medical Center	EHR	280
10	SIDIAP	The Information System for Research in Primary Care	EHR (primary care linked to inpatient)	16,978
11	HIRA COVID	Health Insurance Review & Assessment Service (HIRA), COVID-19 database (South Korea)	medical claims	6,223

Supplementary Table 4. Data summary of all the collaborative sites. CCI: Charlson comorbidity index; Race (NHW): non-hispanic white; COPD: Chronic obstructive pulmonary disease; LOS: length of stay. The reference groups for age is 18-65, for CCI is 0-1, for gender is female, for race is others, and for admission is Q1 (the first quarter of 2020). * : Race may be subject to missing values in some of the sites, and thus was not used in the analysis.

	UHG.NE No. (%)	UHG.S No. (%)	UHG.MW No. (%)	UHG.W No. (%)	OneFlorida No. (%)	STARR No. (%)	HIRA COVID No. (%)	CUIMC No. (%)	Optum EHR No. (%)	CCAE No. (%)	MDCR No. (%)	SIDIAP No. (%)	Optum COVID No. (%)	TRDW No. (%)
Total	12178 (100)	20565 (100)	12717 (100)	2296 (100)	2626 (100)	995 (100)	6223 (100)	3289 (100)	6114 (100)	18185 (100)	4024 (100)	16978 (100)	14139 (100)	280 (100)
Age (65-80)	5919 (48.6)	11174 (54.3)	6295 (49.5)	1183 (51.5)	696 (26.5)	298 (29.9)	876 (14.1)	933 (28.4)	1804 (29.5)	407 (2.2)	1986 (49.4)	5209 (30.7)	3566 (25.2)	85 (30.4)
Age (80+)	5199 (42.7)	6269 (30.5)	5171 (40.7)	908 (39.5)	345 (13.1)	65 (6.5)	327 (5.3)	477 (14.5)	1048 (17.1)	0 (0)	1991 (49.5)	3705 (21.8)	1705 (12.1)	36 (12.9)
CCI (2-5)	4444 (36.5)	7057 (34.3)	4442 (34.9)	862 (37.5)	800 (30.5)	281 (28.2)	2093 (33.6)	813 (24.7)	179 (2.9)	5895 (32.4)	977 (24.3)	4811 (28.3)	3953 (28)	62 (22.1)
CCI (5+)	4297 (35.3)	8487 (41.3)	4816 (37.9)	773 (33.7)	890 (33.9)	339 (34.1)	782 (12.6)	1350 (41)	127 (2.1)	4043 (22.2)	2687 (66.8)	1461 (8.6)	2273 (16.1)	114 (40.7)
Gender (male)	5704 (46.8)	9045 (44)	5805 (45.6)	1071 (46.6)	1088 (41.4)	481 (48.3)	2396 (38.5)	1532 (46.6)	3081 (50.4)	9322 (51.3)	1913 (47.5)	9302 (54.8)	7297 (51.6)	161 (57.5)
*Race (NHW)	9317 (76.5)	13496 (65.6)	10222 (80.4)	2048 (89.2)	877 (33.4)	503 (50.6)	0 (0)	937 (28.5)	3171 (51.9)	0 (0)	0 (0)	0 (0)	6411 (45.3)	134 (47.9)
Admission (Q2)	6940 (57)	8316 (40.4)	5774 (45.4)	1055 (45.9)	843 (32.1)	613 (61.6)	362 (5.8)	1655 (50.3)	4185 (68.4)	7543 (41.5)	2203 (54.7)	6968 (41)	10724 (75.8)	210 (75)
Admission (Q3)	4465 (36.7)	11713 (57)	6646 (52.3)	1142 (49.7)	1783 (67.9)	310 (31.2)	0 (0)	878 (26.7)	0 (0)	8085 (44.5)	1017 (25.3)	0 (0)	1301 (9.2)	38 (13.6)
Cancer	2580 (21.2)	3993 (19.4)	2596 (20.4)	443 (19.3)	453 (17.3)	323 (32.5)	357 (5.7)	628 (19.1)	68 (1.1)	1957 (10.8)	1518 (37.7)	2274 (13.4)	1311 (9.3)	57 (20.4)
COPD	2745 (22.5)	5622 (27.3)	3138 (24.7)	530 (23.1)	796 (30.3)	88 (8.8)	777 (12.5)	436 (13.3)	89 (1.5)	1402 (7.7)	1390 (34.5)	1315 (7.7)	1500 (10.6)	54 (19.3)
Hypertension	9587 (78.7)	16994 (82.6)	9800 (77.1)	1605 (69.9)	1948 (74.2)	491 (49.3)	1541 (24.8)	1975 (60)	313 (5.1)	10710 (58.9)	3653 (90.8)	4817 (28.4)	7896 (55.8)	157 (56.1)
Hyperlipidemia	7552 (62)	12936 (62.9)	7314 (57.5)	1152 (50.2)	1567 (59.7)	443 (44.5)	2528 (40.6)	1433 (43.6)	264 (4.3)	9947 (54.7)	3454 (85.8)	3025 (17.8)	5804 (41)	133 (47.5)
Kidney disease	3974 (32.6)	8048 (39.1)	4804 (37.8)	838 (36.5)	1368 (52.1)	297 (29.8)	776 (12.5)	1289 (39.2)	190 (3.1)	4684 (25.8)	2453 (61)	3700 (21.8)	5096 (36)	124 (44.3)
Obesity	1407 (11.6)	2740 (13.3)	1455 (11.4)	263 (11.5)	1039 (39.6)	465 (46.7)	14 (0.2)	1606 (48.8)	3608 (59)	9729 (53.5)	1580 (39.3)	7766 (45.7)	6811 (48.2)	137 (48.9)
Heart disease	7286 (59.8)	12270 (59.7)	7766 (61.1)	1264 (55.1)	964 (36.7)	370 (37.2)	843 (13.5)	1610 (49)	236 (3.9)	6318 (34.7)	3267 (81.2)	4457 (26.3)	6376 (45.1)	154 (55)
Diabetes	5045 (41.4)	10120 (49.2)	5275 (41.5)	887 (38.6)	1302 (49.6)	233 (23.4)	1412 (22.7)	1228 (37.3)	193 (3.2)	6576 (36.2)	2167 (53.9)	3046 (17.9)	5049 (35.7)	99 (35.4)
LOS (mean, sd)	9.6 (13.3)	8.6 (10.4)	7.9 (9.7)	7.9 (10.2)	11 (14.9)	5.1 (6.4)	16.1 (11)	9.6 (18.4)	7.8 (7.9)	8.4 (13.2)	11.9 (16.9)	6.3 (9.4)	9.5 (11.3)	9.2 (13.1)

Supplementary Table 5. The random effects selection based on the likelihood ratio test. The covariates are sequentially tested (forward selection) starting from the model with random intercept only. All the covariates except obesity are tested as having significant random effects. The reference groups for age is 18-65, for CCI is 0-1, for gender is female, for admission is Q1 (the first quarter of 2020).

Covariate added	Likelihood ratio	p-value	Estimated variance component
Intercept	NA	NA	7.47
Kidney disease	478	<0.001	1.57
Heart disease	234.7	<0.001	1.19
CCI (5+)	176	<0.001	7.75
Admission (Q2)	161.4	<0.001	1.62
CCI (2-5)	82.3	<0.001	2.22
Age (80+)	74.4	<0.001	2.21
Admission (Q3)	65.6	<0.001	2.35
Gender (male)	41	<0.001	0.29
Age (65-80)	31.8	<0.001	0.68
COPD	25.7	<0.001	0.43
Hyperlipidemia	24.6	<0.001	0.38
Hypertension	13.6	<0.001	0.16
Diabetes	9.4	0.001	0.3
Cancer	7.7	0.003	0.26
Obesity	0	0.5	NA

Supplementary References

1. Datta, S., Posada, J., Olson, G., Li, W., O'Reilly, C., Balraj, D., Mesterhazy, J., Pallas, J., Desai, P. and Shah, N., 2020. A new paradigm for accelerating clinical data science at Stanford Medicine. *arXiv preprint arXiv:2003.10534*.
2. Optum® de-identified COVID-19 Electronic Health Record dataset (2007-2020).
3. Rho Y, Cho DY, Son Y, Lee YJ, Kim JW, Lee HJ, et al. COVID-19 International Collaborative Research by the Health Insurance Review and Assessment Service Using Its Nationwide Real-world Data: Database, Outcomes, and Implications. *J Prev Med Public Health*. 2021;54(1):8-16.
4. Debray, T. P. A., Koffijberg, H., Vergouwe, Y., Moons, K. G. M. & Steyerberg, E. W. Aggregating published prediction models with individual participant data: a comparison of different approaches. *Stat. Med.* **31**, 2697–2712 (2012).
