# Supplementary material for: DLMM as a lossless one-shot algorithm for collaborative multi-site distributed linear mixed models
Source: Nat Commun. 2022 Mar 30;13:1678. doi: 10.1038/s41467-022-29160-4 (PMC8967932; doi:10.1038/s41467-022-29160-4)
Supplement: Supplementary file 2 — Reporting Summary [file 41467_2022_29160_MOESM2_ESM.pdf]

nature research

Corresponding author(s): Yong Chen

Last updated by author(s): 02/23/2022

Reporting Summary

Nature Research wishes to improve the reproducibility of the work that we publish. This form provides structure for consistency and transparency in reporting. For further information on Nature Research policies, see our Editorial Policies and the Editorial Policy Checklist.

Statistics

For all statistical analyses, confirm that the following items are present in the figure legend, table legend, main text, or Methods section.

n/a	Confirmed	
☐	☒	The exact sample size (*n*) for each experimental group/condition, given as a discrete number and unit of measurement
☐	☒	A statement on whether measurements were taken from distinct samples or whether the same sample was measured repeatedly
☐	☒	The statistical test(s) used AND whether they are one- or two-sided<br>*Only common tests should be described solely by name; describe more complex techniques in the Methods section.*
☐	☒	A description of all covariates tested
☐	☒	A description of any assumptions or corrections, such as tests of normality and adjustment for multiple comparisons
☐	☒	A full description of the statistical parameters including central tendency (e.g. means) or other basic estimates (e.g. regression coefficient) AND variation (e.g. standard deviation) or associated estimates of uncertainty (e.g. confidence intervals)
☐	☒	For null hypothesis testing, the test statistic (e.g. *F*, *t*, *r*) with confidence intervals, effect sizes, degrees of freedom and *P* value noted<br>*Give P values as exact values whenever suitable.*
☒	☐	For Bayesian analysis, information on the choice of priors and Markov chain Monte Carlo settings
☒	☐	For hierarchical and complex designs, identification of the appropriate level for tests and full reporting of outcomes
☐	☒	Estimates of effect sizes (e.g. Cohen's *d*, Pearson's *r*), indicating how they were calculated

Our web collection on statistics for biologists contains articles on many of the points above.

Software and code

Policy information about availability of computer code

Data collection	Part of the data (aggregated data from CCAE, MDCR, STARR, CUIMC, Optum EHR, TRDW, SIDIAP, HIRA COVID) are collected by the OHDSI DistributedLMM protocol (https://github.com/ohdsi-studies/DistributedLMM) and processed by J.R.. Other data (UHG by M.N.I., OneFlorida by Z.C., Optum COVID by Y.Z.) are processed by the corresponding co-authors. All the aggregated data are sent to the first author (C.L.) for final analysis. The EHR/claims data are proprietary and are not publicly accessible due to restricted user agreement. The detailed data description and IRB statements of each data sets are in the Supplementary Notes. For replication purpose, the dataset from the OneFlorida consortium can be obtained by contacting Dr. Jiang Bian (email: bianjiang@ufl.edu) upon the completion of the data usage agreement.
Data analysis	The R code for running DLMM is wrapped in the R (version >= 3.5.0) package "pda" version 1.0-2, available at CRAN (https://CRAN.R-project.org/package=pda) or github (https://github.com/Penncil/pda). A separate documentation for running DLMM using simulated data is at https://github.com/Penncil/DLMM

For manuscripts utilizing custom algorithms or software that are central to the research but not yet described in published literature, software must be made available to editors and reviewers. We strongly encourage code deposition in a community repository (e.g. GitHub). See the Nature Research guidelines for submitting code & software for further information.

Data

Policy information about availability of data

All manuscripts must include a data availability statement. This statement should provide the following information, where applicable:

- Accession codes, unique identifiers, or web links for publicly available datasets
- A list of figures that have associated raw data
- A description of any restrictions on data availability

Part of the data (aggregated data from CCAE, MDCR, STARR, CUIMC, Optum EHR, TRDW, SIDIAP, HIRA COVID) are collected by the OHDSI DistributedLMM protocol

(https://github.com/ohdsi-studies/DistributedLMM) and processed by J.R.. Other data (UHG by M.N.I., OneFlorida by Z.C., Optum COVID by Y.Z.) are processed by the corresponding co-authors. All the aggregated data are sent to the first author (C.L.) for final analysis. The EHR/claims data are proprietary and are not publicly accessible due to restricted user agreement. The detailed data description and IRB statements of each data sets are in the Supplementary Notes. For replication purpose, the dataset from the OneFlorida consortium can be obtained by contacting Dr. Jiang Bian (email: bianjiang@ufl.edu) upon the completion of the data usage agreement.

Field-specific reporting

Please select the one below that is the best fit for your research. If you are not sure, read the appropriate sections before making your selection.

- [x] Life sciences
- [ ] Behavioural & social sciences
- [ ] Ecological, evolutionary & environmental sciences

For a reference copy of the document with all sections, see nature.com/documents/nr-reporting-summary-flat.pdf

Life sciences study design

All studies must disclose on these points even when the disclosure is negative.

Sample size	The project leaders (Y.C., M.S. and J.R.) post the research protocol (https://github.com/ohdsi-studies/DistributedLMM) to the OHDSI forum to recruit collaborators. Data of 120,609 COVID-19 patients that admitted in the first three quarters of 2020 are extracted from 11 collaborative data sources worldwide. These observational data are considered as a representative sample of COVID-19 hospitalization patients with probable site-level heterogeneity.
Data exclusions	The patients with less than 180 days of observation time prior to the index date (i.e. date of hospitalization), or aged under 18 are excluded. This exclusion criteria is pre-established.
Replication	The UnitedHealth Group Clinical Discovery Portal (UHG) data (N=47,756)is used to demonstrate the lossless property of the DLMM algorithm. This is confirmed as estimation of fixed and random effects are identical between DLMM and pooled LMM, see Figure 3. The association findings (i.e. the fixed effects of patients' characteristics on length of stay) are confirmed using the international data (N=120,609), see Figure 6 for a comparison.
Randomization	No randomization in this study as this is an association study using observational data (EHR and medical claims data sets).
Blinding	Not relevant as this is an observational study.

Reporting for specific materials, systems and methods

We require information from authors about some types of materials, experimental systems and methods used in many studies. Here, indicate whether each material, system or method listed is relevant to your study. If you are not sure if a list item applies to your research, read the appropriate section before selecting a response.

Materials & experimental systems

n/a	Involved in the study	
x		Antibodies
x		Eukaryotic cell lines
x		Palaeontology and archaeology
x		Animals and other organisms
	x	Human research participants
x		Clinical data
x		Dual use research of concern

Methods

n/a	Involved in the study	
x		ChIP-seq
x		Flow cytometry
x		MRI-based neuroimaging

Human research participants

Policy information about studies involving human research participants

Population characteristics	- demographics including Age, gender, race (only in UHG data),<br>- clinical characteristics include a history of cancer, chronic obstructive pulmonary disease (COPD), heart disease, hypertension, hyperlipidemia, diabetes, kidney disease, obesity, and the Charlson comorbidity index (CCI) score,<br>- admission date (categorized as Q1, Q2, or Q3, i.e., admission in the first, second, or third quarter of 2020, respectively).
Recruitment	The project leaders post the research protocol (https://github.com/ohdsi-studies/DistributedLMM) to the OHDSI forum to recruit participating sites. The collaborative investigators that committed to the project extract the patient sample from their databases. The self-selection bias is unlikely as the databases involved are large-scale EHR or administrative claims data and the inclusion of patients are based on a predefined protocol.
Ethics oversight	This is a multi-center study. The detailed IRB approval from multiple organizations are available at page 3-6 of the Supplementary Materials.

Note that full information on the approval of the study protocol must also be provided in the manuscript.
